# *Fusarium* Keratitis—Review of Current Treatment Possibilities

**DOI:** 10.3390/jcm10235468

**Published:** 2021-11-23

**Authors:** Marek Szaliński, Aleksandra Zgryźniak, Izabela Rubisz, Małgorzata Gajdzis, Radosław Kaczmarek, Joanna Przeździecka-Dołyk

**Affiliations:** 1 Department of Ophthalmology, Wroclaw Medical University, ul. Borowska 213, 50-556 Wrocław, Poland; marek.szalinski@umed.wroc.pl (M.S.); gosiagajdzis@gmail.com (M.G.); radoslaw.kaczmarek@umed.wroc.pl (R.K.); joanna.przezdziecka-dolyk@pwr.edu.pl (J.P.-D.); 2 Clinic of Ophthalmology, University Teaching Hospital, ul. Borowska 213, 50-556 Wrocław, Poland; 3 Okulus Ophthalmology Clinic, ul. Śródmiejska 34, 62-800 Kalisz, Poland; izabela.rubisz@gmail.com; 4 Department of Optics and Photonics, Wroclaw University of Science and Technology, Wyb. Stanisława Wyspiańskiego 27, 50-370 Wrocław, Poland

**Keywords:** *Fusarium* keratitis, *Fusarium* keratitis treatment, natamycin, voriconazole, amphotericin B

## Abstract

In many parts of the world, fungi are the predominant cause of infectious keratitis; among which, *Fusarium* is the most commonly isolated pathogen. The clinical management of this ophthalmic emergency is challenging. Due to the retardation of the first symptoms from an injury and the inability to differentiate fungal from bacterial infections based on clinical symptoms and difficult microbial diagnostics, proper treatment, in many cases, is postponed. Moreover, therapeutical options of *Fusarium* keratitis remain limited. This paper summarizes the available treatment modalities of *Fusarium* keratitis, including antifungals and their routes of administration, antiseptics, and surgical interventions.

## 1. Introduction

*Fusarium* keratitis is a severe ocular infection and a common cause of monocular blindness in the tropical and subtropical areas of the world. The prevalence of *Fusarium* keratitis is difficult to establish. The annual prevalence of fungal keratitis is estimated to be 1,051,787 cases globally, with the highest estimated amount of incidences in Asia and Africa and the lowest in Europe [1]. While filamentous fungi are the main cause of fungal keratitis in tropical and subtropical climates, yeasts dominate in temperate climates [1,2]. Among globally reported cases of fungal keratitis, filamentous fungus *Fusarium* spp. is the most frequently isolated cause [1].

*Fusarium* spp. is widely distributed in the environment, especially in soil and plants. The main risk factors for *Fusarium* keratitis are trauma from organic matter, contact-lens use, ocular surgeries, ocular surface disease, together with intensive use of steroids [3].

The pathogenesis of *Fusarium* keratitis depends on the characteristics of the individual strain as well as the immunological response of the host [4]. The ability to form biofilm by *Fusarium* species has been investigated in a number of experimental models. *Fusarium* species can produce biofilm in vitro, as well as in contact lens models, and in vivo on the infected cornea [5,6,7,8]. Biofilm is considered a key to *Fusarium* pathogenicity and contributes to its resistance to antifungals [8,9]. In addition, enzymes, such as carboxypeptidase and aminopeptidase, as well as mycotoxins, such as fusaric acid, moniliformin, or fumonisin B1, which are secreted by *Fusarium* strains, play an important role in its virulence [10,11]. Regarding the host immunological response, it was demonstrated that *Fusarium* strains induce macrophage activation, infiltration by polymorphonuclear leukocytes, and increase in the levels of cytokines such as IL-1β, IL-8, IL-17, and TNF-α [12,13]. Increases in the activities of different enzymes, such as myeloperoxidase, inducible nitric oxide synthase, and matrix metalloproteinases, have also been reported [14,15]. The host response is mediated by several receptors, including toll-like-2 and 4 and vitamin D receptors [16,17]. Furthermore, *Fusarium* was reported to induce changes in the protein profile of the host’s tears [18]. It is suspected that a severe course of *Fusarium* infection may result from an excessive host immune response [12].

**Figure 1:**
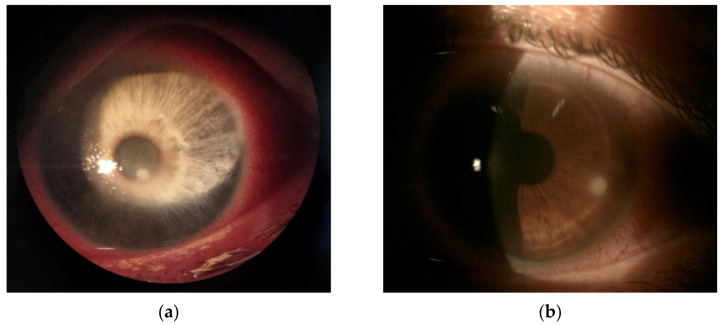
(**a**) An early corneal infiltrate with confirmed etiology of *Stenotrophomonas maltophilia*; (**b**) an early corneal infiltrate with confirmed etiology of *Fusarium* spp.

Because *Fusarium* keratitis, as with other fungal ulcers, can resemble bacterial keratitis, it is not possible to make a firm diagnosis based on clinical presentation (Figure 1). In a large prospective study, features such as serrated margins, raised slough, dry texture, satellite lesions, and color other than yellow, occurred significantly more frequently in fungal ulcers than in bacterial ulcers [3]. It was proven that serrated margins, raised slough, and color other than yellow were statistically independent features of fungal keratitis. The probability of diagnosis was 63% when one of these characteristics was present. If all three were present, the probability increased to 83%. Another study showed feathery margins, raised profile, and dry surface to be associated with fungal keratitis. In this study, *Fusarium* cases were more likely to show feathery margins and colors other than yellow than *Aspergillus* cases. At the same time, ulcers caused by *Aspergillus* more frequently presented hypopyon and raised profiles [19]. In a smaller study, *Fusarium* keratitis was less likely to present with raised slough and ring infiltrates than *Aspergillus* [20]. As in other fungal ulcers, definitive diagnosis of *Fusarium* keratitis is based on smears, cultures, in vivo laser scanning confocal microscope, and PCR, the first two of which are considered the gold standard [21,22].

**Figure 2:**
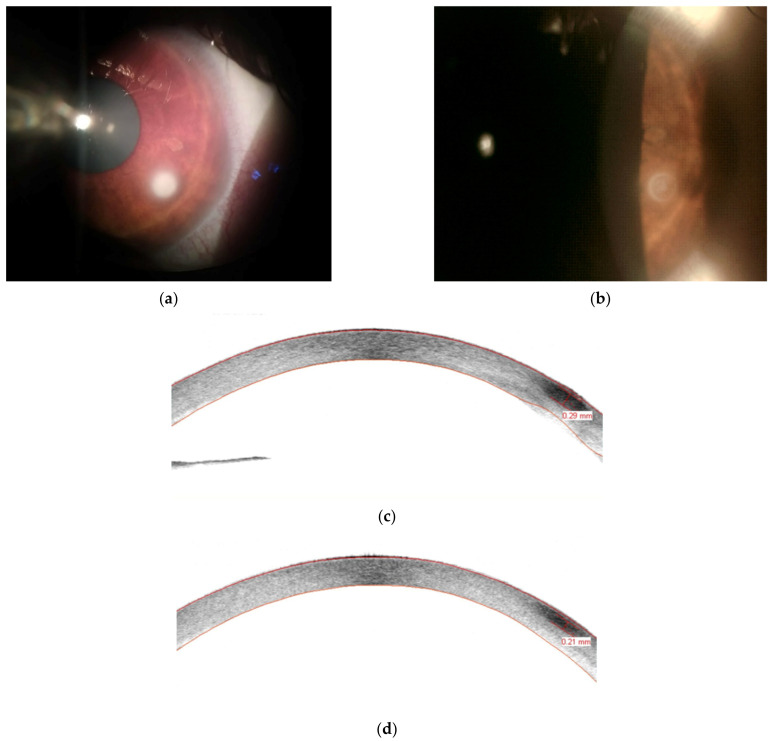
Successful treatment of the small, initial *F. oxysporum SC* infiltrate in a 46-year old contact lens user. (**a**) Opaque, white infiltrate with a cloudy margin in the lower temporal corneal quadrant observed on the fifth day after the first onset of symptoms. Diagnosis was based on the presence of fungal hyphae in confocal microscopy and corneal impression cytology stained with periodic acid–Schiff and hematoxylin–eosin. (**b**) Opacity reduction and sharpening of the infiltrate edges with resolution of the irritation observed on the sixteenth day of treatment with topical 1% voriconazole. AS-OCT scans performed on the second (**c**) and twelfth (**d**) days of treatment. Best-corrected visual acuity remained 20/20 during the entire eight week-long course of illness and treatment.

**Figure 3:**
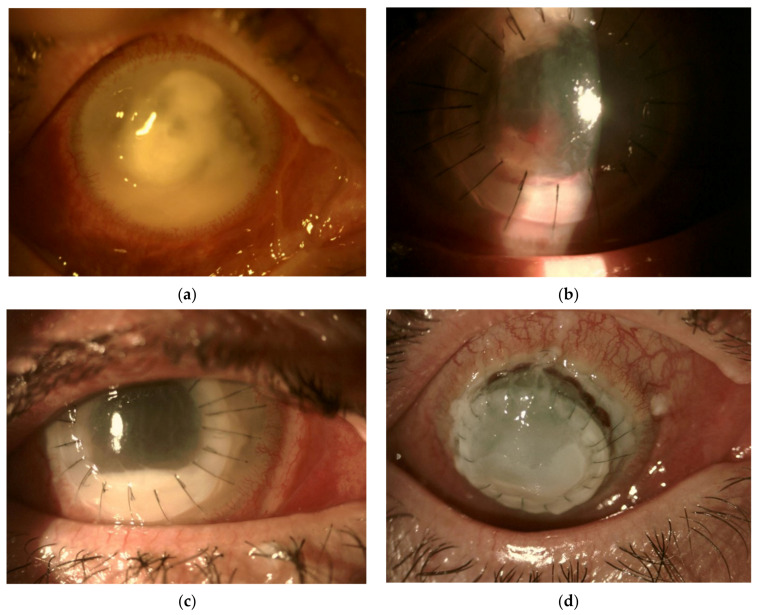
Failure of treatment in a 53-year old patient with severe *Fusarium* spp. keratitis associated with improper use of contact lenses and extended improper initial multi-drug treatment. (**a**) Admission, seven weeks from the onset of the symptoms and after a period of self-administered treatment with the number of topical (fluoroquinolones, gentamycin) and oral antibiotics (cephalosporins), together with topical steroids. Bacterial cultures taken on admission were negative. (**b**) Recurrence of anterior chamber exudate three days after therapeutic penetrating keratoplasty (TPK) performed shortly after admission, due to progressing tissue melting, despite introduced intensive treatment with topical vancomycin, gentamicin, and ceftazidime, as well as fluconazole and ceftazidime intravenously. Probes taken on the tenth day of hospitalization showed growth of mixed flora, including *Corynebacterium* spp., *Staphylococcus aureus*, coagulase-negative *Staphylococcus*, and *Streptococcus mitis*, all of which were susceptible to the treatment. However, progression was observed. (**c**) Twelve days after TPK, fungal cultures taken on admission revealed growth of *Fusarium* spp. Due to hospital internal regulations (amphotericin B was on the list of expensive medications requiring special administrative approval, which could only be obtained after obtaining positive culture), only then was the treatment with topical amphotericin B (AMB) and intravenous, topical, and intracameral voriconazole (VCZ) introduced. (**d**) Thirty days after TPK, melting and luxation of the lens into the anterior chamber requiring lens excision and sutures exchange. Seven weeks after admission, due to continuous progression with corneal tissue melting evisceration was performed.

The course of *Fusarium* infection can vary greatly, depending on the virulence of the subspecies, its sensitivity to antifungal drugs, and the timing of treatment (Figure 2 and Figure 3). It is estimated that, annually, around 100,000 eyes are removed worldwide due to delayed diagnosis and treatment failure of fungal keratitis [1]. As filamentous fungi are associated with the worse prognosis, it could be suspected that *Fusarium* accounts for a large proportion of this number [3].

For these reasons, *Fusarium* keratitis poses a serious ophthalmological emergency. In this review, we summarize the current pharmacological and surgical treatment options.

## 2. Search Strategy

We searched PubMed, Embase, Cochrane Library, Web of Knowledge, and SCOPUS with the keywords “*Fusarium* keratitis”, “fungal keratitis”, “fusarium keratomycosis”, and “mycotic keratitis”, as well as other similar terms. In the preliminary review, we read 747 abstracts, out of which we chose 140 articles to include in this review (for further information about the search strategy and the PICO statement please, review the Supplementary Materials). We included three additional articles after suggestions from reviewers.

## 3. Antifungals

To date, due to the low efficacy of other polyene and azole drugs, natamycin (NAT), amphotericin B (AMB) and voriconazole (VCZ) are the most commonly used drugs in *Fusarium* keratitis [23,24,25]. Regarding allylamines, a retrospective study from China showed promising results of terbinafine used in smaller and shallower ulcers in a group of patients in which *Fusarium* was predominant isolate [26]. Nonetheless, the study of antifungal susceptibility of 426 *Fusarium* isolates collected from patients with fungal keratitis in China showed that *Fusarium* was less sensitive to terbinafine than to NAT and AMB [27].

### 3.1. Mechanisms of Action

**Figure 4:**
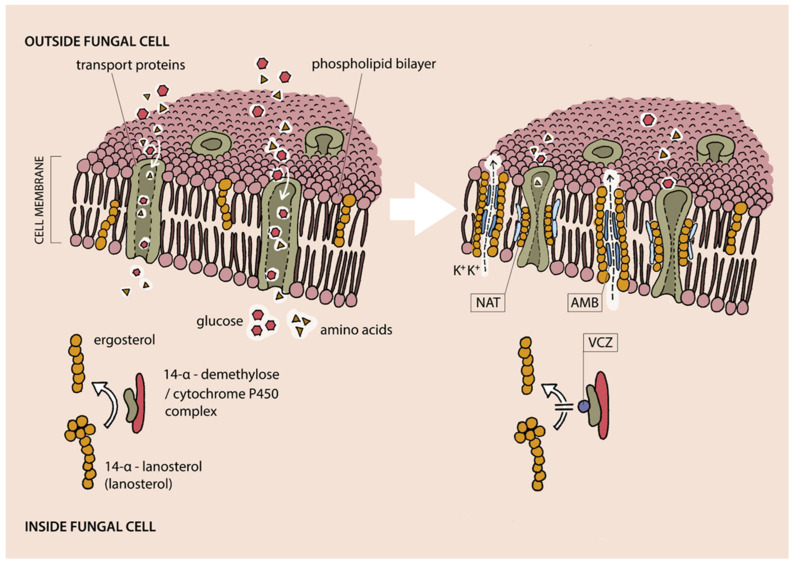
Mechanisms of action of the most commonly used antifungals. AMB—amphotericin B, NAT—natamycin, VCZ—voriconazole. AMB binds to the cell membrane ergosterol, which results in the formation of pores and an increase in the membrane permeability. Binding of NAT to the ergosterol leads to inhibition of glucose and amino acids transport trough adequate membranes transporters. VCZ blocks 14-α-demethylase/cytochrome P450 complex, which leads to a decrease in transformation of 14-α-lanosterol to ergosterol and, as a result, destabilization of the cell membrane.

NAT and AMB are polyenes, whereas VCZ is a triazole. NAT and AMB bind to ergosterol, an amphoteric molecule, which stabilizes fungal cell membrane. In case of AMB, this binding leads to the formation of pores in the cell membrane and the efflux of ions and other substances from inside the cell. The combination of NAT with ergosterol does not affect cell membrane permeability, but it leads to reversible inhibition of amino acids and glucose membrane transport proteins [28,29,30]. The VCZ mechanism of action is attributable to blocking the cytochrome p450 and 14-alpha demethylase complex, which results in a reduction of the ergosterol concentration in the membrane, which leads to destabilization of the membrane [31]. The mechanisms of action of the named antifungals are shown on Figure 4.

### 3.2. Topical Use

#### 3.2.1. Natamycin

We should note that 5% NAT is the only Food and Drug Administration (FDA) approved drug registered for use (Natacyn^®^) in the ocular fungal infections in the United States. Despite the high molecular weight limiting penetration into the deeper ocular tissues and, hence, the need for frequent and prolonged administration, it remains a gold standard for fungal keratitis [32,33,34]. Its wide antifungal spectrum covers the majority of *Fusarium* species and it was shown in a number of in vitro and in vivo studies [23,34]. One meta-analysis suggested that, when compared to other antifungals, NAT was particularly effective in treating initial *Fusarium* keratitis [35]. A Chinese study of 525 patients, among which 19.25% were positive for *Fusarium*, showed significant correlation between size of inhibition zones in susceptibility tests of NAT and clinical outcomes. The larger inhibition zones were associated with the higher rates of cure and the lower rates of evisceration. This was not observed in the case of two other tested antifungals [36]. Nonetheless, NAT used as monotherapy might not be effective in a significant proportion of *Fusarium* cases, even with proven in vitro susceptibility of the isolates. In one study, 46.6% cases required keratoplasty, regardless of the time of presentation, despite *Fusarium* isolates being sensible to NAT [37]. In the Mycotic Ulcer Treatment Trial I, which we describe below, 12.8% of NAT-treated patients either progressed to perforation or required therapeutic keratoplasty [38,39].

Although NAT is the only topical ophthalmic antifungal included on the 22nd WHO Model List of Essential Medicines (2021), it is not licensed in many low- and middle-income countries (LMICs), or in Europe (including the United Kingdom) [40,41,42]. Another problem is its high cost, which, among others, is attributable to the difficult formulation [27,43]. The limited availability of what is currently deemed the most effective drug for *Fusarium* keratitis treatment is, undoubtedly, a serious problem faced by practitioners, especially in LMICs, where the burden of this disease is greatest.

#### 3.2.2. Natamycin vs. Amphotericin B

Similar to NAT, amphotericin B (AMB) is a polyene drug. In in vitro susceptibility tests of *Fusarium* species, minimal inhibitory concentrations (MICs) for AMB were similar or lower than those for NAT [44,45,46,47]. Nonetheless, the most commonly used AMB concentration of 0.15% is much lower than 5% used for NAT, potentially allowing NAT superior bioavailability. Part of a randomized trial on collagen cross-linking in fungal keratitis compared the efficacy of topical 5% NAT to 0.15% AMB. Patients treated with AMB were more likely to have positive cultures at 3 days of follow-up then those treated with NAT. Additionally, the study showed that patients treated with AMB more frequently had epithelial defects; however, the difference was not statistically significant. No difference in ulcer or scar size was found at any follow-ups between the groups [48]. More frequent epithelial defects in the AMB group may be explained by the toxic effects of AMB previously reported in in vitro and in vivo studies, which revealed reduced viability and migration of the epithelial cells leading to the retardation of healing [49,50].

Taking into account the theoretical lower efficacy and possible side effects of AMB, and the lack of robust evidence, NAT is currently considered the first-line treatment for *Fusarium* keratitis in countries where NAT is available. Further research in the form of adequately powered, large randomized controlled trials are required to determine the efficacy and safety of AMB vs. NAT.

Because, in many reports, MICs of AMB were much lower than MICs of voriconazole (VCZ), AMB is likely a better choice than VCZ for first line *Fusarium* keratitis treatment in countries where NAT is not available [24,46,51].

#### 3.2.3. Natamycin vs. Voriconazole

Among the more widely available azoles, *Fusarium* spp. is resistant to fluconazole, itraconazole, and ketoconazole [23,24]. In contrast, a newer second-generation drug, voriconazole, with proven good penetration into the eyeball, has gained popularity in the treatment of filamentous keratitis [52,53].

The well-known Mycotic Ulcer Treatment Trial I (MUTT I), which was conducted in South India, among other filamentous fungal keratitis patients, included 128 (40%) patients with *Fusarium* keratitis [38,54]. Most of the patients in the trial were from rural, tropical regions, with a history of corneal trauma. In this multicenter, double-masked trial, patients were treated with either topical 5% NAT or 1% VCZ administered hourly while awake for one week, and every two hours while awake for another two weeks. In this study, cultures taken on the sixth day were more likely to be positive in a VCZ-treated group than in the NAT-treated group (48% vs. 15%, respectively), with the same pattern in the *Fusarium* subgroup (60% in the VCZ-treated group vs. 8% in the NAT-treated group). The mean best spectacle-corrected visual acuity (BSCVA) at three months was 1.8 lines better in patients treated with NAT than in patients treated with VCZ. The difference was more prominent in the subgroup analysis of *Fusarium* cases, in which the mean BSCVA at three months in the NAT-treated group was 4.1 lines better than in the VCZ-treated group. Additionally, in the *Fusarium* subgroup, out of seven patients with perforations, six were treated with VCZ. According to the results, monotherapy with VCZ for *Fusarium* keratitis was not recommended. In vitro susceptibility testing results of the isolates from this trial were consistent with the in vivo results. *Fusarium* isolates were less susceptible to VCZ than to NAT and significantly less susceptible to VCZ than other fungal isolates [55]. Secondary analysis of MUTT I showed that culture positivity of the corneal scrapes taken on the sixth day of treatment significantly correlates with the worse prognosis [56,57].

MUTT I, along with two other trials, was included in the important systematic review on medical interventions in fungal keratitis made by FlorCruz [38,39,58,59]. While the majority of compared interventions did not show significant clinical differences, comparisons between NAT and VCZ showed superiority of NAT in the treatment of fungal keratitis. This was particularly evident in the reduction of the risk of corneal perforation.

Another randomized trial, also conducted in India, not included in the above-mentioned review, also found NAT to be superior to VCZ in fungal keratitis. Among 118 patients, the study included 29 *Fusarium* keratitis cases. In this study, the percentage of healed or resolving ulcers at the last follow-up was significantly higher in the NAT-treated group than in the VCZ-treated group (89.2% vs. 66.5%, respectively). While visual acuity improvement was significant on both day 7 and the last follow-up in the VCZ-treated group, the improvement of visual acuity was significant at day 7 and highly significant at the final follow-up in the NAT-treated group. In terms of visual acuity improvement, response to NAT was significantly better in patients with *Fusarium* keratitis. This was not observed in the subgroup with *Aspergillus* keratitis [60].

The higher in vitro susceptibility to NAT than to VCZ was shown to be particularly pronounced for the *Fusarium solani*, species known to have worse prognosis than other *Fusarium* isolates [23,61]. In contrary, in the experimental animal model of *Fusarium solani* keratitis, VCZ showed better efficacy than NAT and AMB, in terms of reducing infiltrate size as well as fungal load [62].

Based on the published research—topical NAT appears to be a much better choice than VCZ for first line treatment for *Fusarium* keratitis.

#### 3.2.4. Natamycin vs. Econazole

In some LMICs, where availability to antifungals is limited, econazole is used in fungal keratitis therapy [41,63,64]. In a randomized trial conducted in India, in which *Fusarium* was predominant isolate (64/112), 2% econazole was found to be as effective as 5% NAT in terms of ulcer healing at four weeks [65]. In a study from Saudi Arabia, in which two hundred *Fusarium* isolates were tested for susceptibility to several antifungals, MICs of econazole were higher than MICs of AMB, similar to MICs of VCZ, and lower than MICs of NAT [25]. Some authors insisted on the inclusion of econazole on the WHO Model List of Essential Medicines for East Africa [41].

As it stands, there is limited evidence comparing NAT to econazole, particularly in relation to objective outcome data, such as BSCVA, making treatment recommendations difficult; further research in the form of adequately powered randomized controlled trials is required.

#### 3.2.5. Combination Therapy

In vitro susceptibility testing of ten *Fusarium* isolates from culture-positive keratitis cases showed no synergistic effect of NAT and AMB [66]. This is probably due to similarity in mechanism of action. We found two reports assessing the synergistic effect of NAT and VCZ. While one of them revealed 23.1% synergism in inhibiting *Fusarium* strains, the other showed synergistic effect in 70% of *Fusarium* isolates. NAT and VCZ appear to be a beneficial combination in the treatment of *Fusarium* keratitis [67,68].

We also found an interesting report about a combination of AMB with rifampicin, a drug used for years in trachoma treatment with proven safety. Rifampicin has no antifungal effect when used alone [69]. It was suspected that AMB, which increases the membrane’s permeability, enables penetration of rifampicin into the cell where it blocks the DNA-dependent RNA polymerase subunit B. In vitro activity tests against *F. solani SC* of multiple rifampicin concentrations (4–32 µg/mL) showed synergism with a range from 11.8 to 94.1%. The clinical use of this combination requires further research.

### 3.3. Oral Antifungals

Mycotic Ulcer Treatment Trial II (MUTT II), conducted in South India and Nepal, aimed to assess the efficacy of oral VCZ, used as additional therapy in severe filamentous fungal keratitis (BCVA at enrolment < 20/200) [70]. Initially, patients received topical 1% VCZ in a regimen similar to MUTT I and oral VCZ (a dose of 2 × 400 mg on the first two days and 2 × 200 mg on subsequent days) or placebo. After MUTT I results became available, topical 5% NAT was added to the protocol in both arms. MUTT II showed no benefit of adding oral VRZ to the treatment of advanced mycotic ulcers. In addition, there was an increased incidence of adverse events in the oral VCZ group. A sub-analysis by species suggested a slight beneficial effect in the *Fusarium* subgroup, in terms of lower rates of perforations and therapeutical keratoplasties; however, the difference was not statistically significant [71].

Contrary to MUTT II, a randomized clinical trial conducted in North India, which compared the efficacy of oral VCZ to oral ketoconazole (KCZ) in severe keratitis, showed beneficial effects of oral VCZ [72]. At three months, the oral VCZ-group presented smaller scar sizes and better visual acuity. The study sample was smaller than in MUTT II (50 vs. 240) and the most common species was not *Fusarium* (40%), but *Aspergillus* (48.5%). In another randomized, double-masked trial of deep fungal keratitis, in which the *Fusarium* spp. was a predominant causative species, adding oral KCZ to topical 5% NAT, showed no benefit in treatment [73].

Based on the available randomized trials, the addition of oral voriconazole may be considered in severe cases of fungal keratitis caused by *Fusarium* strains, but there is insufficient evidence for its inclusion in the standard treatment protocols. In each case, the ratio of the potential benefits should be confronted with these adverse events, and a personalized approach for each patient should take into account the comorbidities and other aspects.

### 3.4. Intrastromal Use

In refractory cases of *Fusarium* corneal ulcers, adjunctive intrastromal use of antifungals was proposed in several studies as a measure to achieve higher drug concentrations in the affected tissues. Nonetheless, the experimental and clinical results are still not conclusive.

In an experimental model, Iranian researchers compared the efficacy of topical 5% NAT to topical 1% VCZ and intrastromal voriconazole injections (ISV) in *Fusarium* keratitis in rabbits. Single ISV and topical 5% NAT were similarly effective and superior to topical 1% VCZ [74].

A case series of twenty-five fungal keratitis patients treated in Southeast India included seven *Fusarium* positive cases. Patients were initially treated with topical 5% NAT for two weeks. When no effect or worsening was observed, topical 1% VCZ was added for another two weeks. In case of failure of this therapy, 50 µ/0.1 mL VCZ was injected intrastromally in five points surrounding the infiltrate. If necessary, ISV was repeated up to three times. Eighteen patients were successfully treated. Out of seven treatment failures, six were positive for *Fusarium* [75]. In another three case reports, in total, eight cases of recalcitrant *Fusarium* treatment were described, three of which were failures. In two of these cases, the infiltrates were large (11 × 10 mm and 9 mm) at the time of presentation [76,77,78].

We found three randomized trials on the intrastromal route of antifungal drug administration. The Mycotic Antimicrobial Localized Injection (MALIN) trial conducted in recent years was designed to assess the efficacy of ISV used as an additional treatment to topical 5% NAT, in moderate to severe fungal keratitis. In this study, ISV was given at the time of diagnosis. A total of 19 out of 70 (27.1%) patients were positive for *Fusarium*. This study showed no benefit of ISV in terms of microbial outcomes, visual acuity, the number of perforations, or corneal transplantations needed. Researchers suggested a possible increase in scar size in cases treated with ISV. *Fusarium* cases did not differ from the others in their course [79]. Another study compared topical VCZ to ISV used as an additional treatment in patients who showed no improvement after two weeks of treatment with topical NAT. The group treated with ISV showed significantly worse BSCVA after treatment. This could have resulted from the more central location of the ulcers in the ISV-group than in the topical VZC group (90% vs. 70%, respectively). In this study, out of forty patients, seven were positive for *Fusarium* [80]. Another recently published, prospective randomized clinical trial compared the effects of intrastromal injections of NAT, VCZ, and AMB in patients who, similar to the formerly described trial, showed progression or no improvement on topical 5% NAT therapy administered for two weeks. The study included 24/60 (40%) *Fusarium* positive cases. In all of the groups, more than 90% of patients were healed with the proposed treatment regimen. The mean duration of healing was shorter in the group treated with intrastromal NAT. Higher rates of vascularization were reported in the AMB group [81].

As the evidence for intrastromal application of antifungals is modest, further research, including the meta-analysis of the described trials, is warranted before any treatment recommendations can be made.

### 3.5. Intracameral Injections

Another way of increasing concentration of the drug in the deep corneal tissues might be the application to anterior chamber, which is usually used as adjunctive therapy.

Several cases of successful treatment of *Fusarium* keratitis, with hypopyon, with intracamerally administered AMB 50 µg/0.1 mL used as adjunctive therapy, were published. When needed, injections were repeated up to five times. In one of these case series, intracameral AMB injections were combined with intrastromal AMB applications [82,83]. On the other hand, reports from a randomized clinical trial conducted in India showed no benefit of intracameral AMB injections used additionally to topical natamycin and oral antifungal. In this study, out of 45 cultures, only two showed growth of *F. solani* [84].

In a few cases, intracameral VZC at a dose of 50 or 100 µg/0.1 mL, repeated up to eight times, was reported to be effective in deep *Fusarium* keratitis, in some cases with endophthalmitis [85,86].

The available evidence for this route of administration is scant. Therefore, intracameral injections of AMB and VCZ in treatment of *Fusarium* keratitis should be considered with appropriate caution, possibly, as a last resort treatment before transplant.

### 3.6. Topical, Systemic, and Targeted Therapy Protocol

Although a number of studies on the efficacy of different treatment modalities have been published, to date, there is still a lack of evidence to clearly guide fungal keratitis treatment. Sharma et al. treated 223 cases of fungal keratitis with the so-called “Topical, Systemic, and Targeted Therapy Protocol” [87]. Initially, all cases were treated with topical 5% NAT hourly in the first forty-eight hours, every two hours while awake until complete re-epithelialization, and every four hours for three weeks. If a lack of improvement or worsening were observed on the seventh to tenth days, topical 1% VCZ was added in a frequency similar to NAT. In case of a poor response after another seven to ten days, intrastromal VCZ was administered every seventy-two hours (maximum four injections). Subsequently, 200 mg of oral VCZ or KCZ twice a day were started at the time of diagnosis for large and/or deep infiltrates. Therapeutic keratoplasty was conducted in cases refractory to the treatment and in cases of corneal perforations. The majority of culture proven cases were *Fusarium* in this study (63/149). The general protocol success rate was 79.8%. In the group treated with intrastromal VCZ, the rate was even higher and equaled 89%. Unfortunately, a sub-analysis of particular species was not conduced. Nonetheless, as this is the first attempt to create fungal keratitis treatment regimen, this protocol might be of unique value for clinicians treating *Fusarium* keratitis.

The most commonly used antifungals, their dosages, and methods of administration are summarized in Table 1.

### 3.7. New Antifungals

In the reviewed reports, we found a total of 6 cases describing successful *Fusarium* keratitis treatment with the use of oral posaconazole [88,89,90]. In the case caused by *F. solani*, in which keratitis evolved into endophthalmitis, posaconazole was also administered topically. An Iranian report on fungal susceptibility patterns to azoles revealed excellent posaconazole susceptibility of *F. solani*, *F. oxysporum*, *F. fujikuroi*, *F. falciforme*, and *F. proliferatum*, but not *F. keratoplasticum* [91]. Nonetheless, the reports from the Netherlands and Spain showed high resistance of all tested *Fusarium* species to posaconazole [24,51]. The use of posaconazole in *Fusarium* keratitis requires further research.

Recently, in vitro study of 18 *Fusarium* isolates (FSSC, FOSC, FCSC, FIESC, FFSC) showed promising results of luliconazole [92]. This new imidazole antifungal had the lowest MIC_90_s (0.06 μg/mL) among 11 substances tested. Moreover, MIC_90_ values were much lower than that recorded for NAT (4 μg/mL) and VCZ (8 μg/mL). The in vivo use of this novel drug in *Fusarium* keratitis is still to be tested.

Although caspofungin, a relatively new echinocandin agent, is mentioned in some reviews as an alternative antifungal treatment in some keratitis cases, we found no report of the *Fusarium* case treated with this drug [93].

### 3.8. New Drug Delivery Possibilities

As poor antifungal penetration is a significant limitation of the therapy, the research is ongoing in order to improve its bioavailability. Experimental in vitro and in vivo use of a drug delivery system based on the polymeric vector of econazole revealed highly enhanced corneal penetration and perfect antifungal activity on *F. solani* isolates [94]. Chinese researchers designed and fabricated hybrid natural hydrogels with VCZ microspheres that could continuously release the drug for up to seven days and excellently inhibit *F. solani* growth [95]. To improve tissue penetration of NAT, it was conjugated with cell penetrating peptides [96]. These synthetized nanocarriers showed complete in vitro inhibition of *F. solani* growth. In a murine model of *Fusarium dimerum* keratitis NAT, conjugated with a cell penetrating peptide was fully effective in 44% of animals, while in the group treated with classic NAT suspension, it was only 13% [97]. The ongoing research on AMB nanosuspensions is also promising [98].

Although these treatment modalities are promising, further research is required to compare them in vivo to current standard therapies.

## 4. Antiseptics

### 4.1. Chlorhexidine—An Old Novel Cure

In the late 1990s, some promising results of the use of 0.2% chlorhexidine (CHX) in fungal (mostly *Aspergillus* and *Fusarium*) keratitis were published [99,100].

From the more recent publications, we chose two interesting reports by Oliveira dos Santos et al. In the first study, researchers assessed in vitro antifungal susceptibility of 98 *Fusarium* isolates collected from patients with keratitis in the Netherlands and Tanzania [101]. Researchers compared MICs of CHX to MICs of seven antifungals and showed that the inhibiting effect of CHX was broad, but not superior to AMB, NAT, and VCZ. However, fungicidal effect of CHX was found in 90% of the *F. oxysporum* isolates and 100% of the *F. solani* at much lower concentrations than 0.02–0.2%, which are used in clinical practices. The fungicidal activity of other tested substances was lower than activity of CHX. As proof of the clinical significance of the above mentioned results, Oliveira dos Santos et al. published a case series of four patients with culture proven *Fusarium* keratitis, with small infiltrates that were successfully treated with 0.02% CHX alone [102]. The study showed good susceptibility of the isolated *Fusarium* strains to CHX as well as the high CHX fungicidal effect.

In clinical practice, chlorhexidine is probably more often concerned as an adjunctive therapy. One study reported good synergistic antifungal effects of CHX and VCZ, but not of CHX and NAT tested in vitro and in vivo (*G. mellonella* model) on the *F. solani* and *oxysporum* strains collected from infected corneas, skin, nails, and auditory canals [103]. A pilot study from Uganda, in which CHX was used as a adjunctive therapy in recalcitrant fungal keratitis not responding to monotherapy with 5% NAT, among others, covered three cases of *Fusarium* spp. keratitis, of which only one was successfully treated [104]. In one of two other cases that ended up with eviscerations, perforation was found at presentation.

Several publications reported the toxic effects of chlorhexidine observed on the cornea. Time and concentration-dependent toxicity was shown in an in vitro study [105]. Cases of keratitis and permanent corneal opacifications resulting from misuse of CHX antiseptic solutions were described. However, the concentrations of CHX were much higher than those used in ophthalmic solutions (4–5% vs. 0.02–0.2%). In addition, the solutions were alcoholic and contained detergents, unlike the aqueous solutions used to treat fungal keratitis [106,107]. In a large retrospective case series and a randomized clinical trial, in which aqueous CHX was used as an ocular antisepsis prior to intravitreal injections, no toxicity was reported [108,109]. We found one case report in which toxic keratitis associated with the use of the 0.02% solution was suspected [110]. An anaphylactic shock after the topical ophthalmic administration was reported in Japan [111].

Clinical use of CHX in *Fusarium* keratitis warrants further research. The two ongoing randomized trials may bring some practical information for the clinicians. A trial conducted in Nepal aims to prove non-inferiority of topical 0.2% CHX to topical 5% NAT in fungal keratitis [112]. A study comparing treatment of fungal keratitis with topical 5% NAT alone to treatment combination of 5% NAT and 0.2% CHX is underway in East Africa [113].

### 4.2. Povidone Iodine

We found one case of *Fusarium oxysporum* keratitis successfully treated with 1% povidone iodine solution in Senegal [114]. Nonetheless, a pilot study, which compared efficacy of 5% NAT to 0.5% povidone iodine in a *Fusarium solani* animal model, showed no benefit of the latter [115]. Two other studies on susceptibility revealed povidone iodine as ineffective in fungal keratitis [116,117].

### 4.3. Ophthalmic Preservatives

A German case series described three cases of *Fusarium* keratitis treatment with topical 0.02% polyhexamethylene biguanide (PHMB) [118]. Two cases of refractory *Fusarium solani* keratitis resolved on treatment with topical and systemic antifungals with the addition of topical 0.02% polyhexamethylene biguanide (PHMB). In the third case, the patient was primary suspected for *Acanthamoeba* and treated with topical 0.02% polyhexamethylene biguanide (PHMB) and propamidine isethionate alone. Culture showed the growth of *Fusarium* spp. and a confocal scanning microscope revealed the presence of filamentous structures. Subsequently, symptoms of the keratitis resolved without the addition of any antifungal. Researchers assessed MICs of PHMB and propamidine isethionate in isolates from the described cases as well as in five other *Fusarium* isolates (*solani*, *proliferatum*, and *oxysporum*) from other keratitis patients. In all cases, the isolates were susceptible to PHMB and highly resistant to propamidine isethionate.

As PHMB is cheap and widely available, studies comparing its efficacy to classical antifungal drugs are highly anticipated.

## 5. Crosslinking

Collagen crosslinking (CXL), a therapeutic option using riboflavin activated with ultraviolet A light, was originally used to treat keratoconus. Due to its anti-inflammatory and antimicrobial effects, as well as enhancing corneal tissue resistance to enzymatic damage, CXL has also been used in infectious keratitis, especially with corneal melting. Although the protocol is the same, in this indication name, ‘photoactivated chromophore for infectious keratitis-corneal collagen cross-linking’ (PACK-CXL) is used [119,120,121].

Experimental in vitro, in vivo and ex vivo studies in animal and human models have shown confounding results in terms of inhibition of *Fusarium* (species *solani* and *oxysporum* were used in two separate studies) growth, as well as changing levels of metalloproteinases, prevention of morphological changes in corneal tissue, and suppressing the progression of fungal keratitis [122,123,124,125,126]. A randomized clinical trial to evaluate the efficacy of CXL in refractory deep stromal fungal keratitis had to be discontinued with only thirteen patients enrolled due to the strong suspicion that CXL promotes the occurrence of corneal perforations [127]. However, in this trial, all *Fusarium* positive patients, one in the study group (topical treatment with CXL), two in the control group (topical treatment only), healed completely with scarring. Another trial randomized one hundred eleven patients with moderate fungal keratitis into four groups (topical 5% NAT alone, topical 5% NAT with CXL, topical 0.15% AMB alone, and topical 0.15% AMB with CXL) [48]. *Fusarium* was the most common fungal species and grew in cultures collected from 45 patients. No benefit of CXL was demonstrated. CXL had no effect on microbiological outcomes, infiltration or scar size, degree of epithelialization, or incidence of adverse events, such as perforation or need for keratoplasty. Furthermore, negative influence on visual acuity was suspected. It has been suggested that the results of the study may have been distorted due to miscalculation of the sample size, inadequate outcome measures, and an inappropriate PACK-CXL protocol [128,129]. In contrast to the above-mentioned studies, in a randomized study of 41 patients (only nine *Fusarium*-positive cases enrolled) CXL used as adjuvant therapy to topical medical treatment had a positive effect on healing of the infiltrate, as well as length and intensity of the treatment [130]. Positive effects were also reported by clinicians from Columbia, who successfully treated a case of severe marginal *Fusarium* spp. with imminent perforation using novel surgical technique of simultaneous therapeutical keratoplasty and PACK-CXL [131].

Based on the available scientific evidence, the use of PACK-CXL in *Fusarium* keratitis, is, for now, questionable, and requires further research, including adequately powered randomized controlled trials.

Interestingly, in vitro study on *Fusarium solani* and two other fungal isolates showed growth inhibiting effects of the photodynamic therapy (PDT) using another chromophore (rose bengal excited with green light) [132]. In a recently published pilot study on rose bengal photodynamic antimicrobial therapy (RB-PDAT) used in progressive microbial keratitis unresponsive to standard treatment, out of eighteen enrolled patients, four were positive for *Fusarium* [133]. Two of them, who had history of contact-lens use, recovered after RD-PDT. In two others with much larger infiltrates at the time of RD-PDT, therapeutic keratoplasty had to be conducted in the follow-up; one of them further underwent evisceration. We also found an interesting case report of contact lens-associated *F. keratoplasticum* keratitis from Florida [134]. In this case, when in vitro susceptibility tests revealed intermediate resistance to NAT and high resistance to AMB, VCZ, and fluconazole, riboflavin CXL and RB-PDAT were tested in vitro. Only RB-PDAT resulted in the inhibition of fungal growth. These results were consistent with clinical course. Despite topical NAT, oral fluconazole, and intrastromal AMB progression was observed. Conversely, the use of RD-PDAT resulted in successful recovery.

Although the use of RD-PDT in *Fusarium* keratitis appears promising, it still requires further research.

## 6. Argon Laser

In a case series of two patients with *Fusarium* keratitis refractory to topical antifungals, an argon laser, used as an adjunctive therapy, promoted resolution of the infiltrates. Thermal effects and damage to the corneal epithelium that enabled better penetration of antifungals were suggested as possible mechanisms of action [135].

## 7. Therapeutic Keratoplasty

When other treatment modalities fail, therapeutic keratoplasty is a chance to save the eye and preserve vision. Currently, depending on whether or not Descemet’s membrane is infected, therapeutic penetrating keratoplasty (TPK) or therapeutic lamellar keratoplasty (TLK) are used.

In an Indian study involving 198 eyes (31 cases of *Fusarium*)—fungal infection was eradicated in 89.9% of patients who underwent TPK [136]. In the remaining patients, the mean time to relapse was 15 ± 9.3 days (range, 1–28 days), and the larger size of the infiltrate and larger size of the graft were risk factors for recurrence (*p* = 0.007 and *p* = 0.02). While the median graft survival rate was 5.9 months, the grafts <8 mm had better survival than those with >8 mm (*p* = 0.026). In a study of 899 patients, who underwent TPK or TLK, treatment with steroids before surgery, hypopyon, perforation, limbus, and/or lens involvement were the most commonly reported risk factors for recurrence. Infection recurred in 6.34% of patients and there was no statistically significant difference in its frequency between the TPK and TLK group [137]. In a trial, which, among others, included 905 *Fusarium* cases, infection recurred in 68 of them [138]. The rates of recurrence were similar to other fungal species. This study suggested that regrafts should not be delayed longer than 3–5 days in case of recurrence and failure of topical treatment.

Based on this research, it is important to note the point at which keratoplasty should be performed. In view of the increased risk of recurrent infection and graft rejection associated with the size of the infiltration and the degree of fungal penetration into the eyeball, it seems that the decision to transplant should not be delayed. Apart from medical reasons, a small study from Taiwan suggested that early keratoplasty in the treatment of moderate (infiltrates between 3 to 6 mm in diameter and depth of infiltration limited to anterior 2/3) *Fusarium* keratitis enabled shorter hospital stays and lowered treatment costs [139]. We found no reports on long-term cost-effectiveness of TPK.

In the above-mentioned studies, topical antifungal therapy was continued postoperatively, whereas steroid immunosuppression was postponed for at least ten days after surgery and introduced only in cases where there was no sign of recurrence. This is currently the most common approach. We found promising results of using topical 0.5% and 0.1% cyclosporin A (CsA) instead of steroids in early postoperative treatment after TPK in fungal keratitis [140,141]. In some studies, the combination of azoles with CsA showed synergistic fungicidal effect [142,143].

## 8. Conclusions

*Fusarium* keratitis is very difficult to manage. The course of the infection can vary greatly depending on the virulence of the strains, its susceptibility to the antifungals and the time of diagnosis. Clinicians treating ocular *Fusarium* infections are faced with common resistance of isolates and poor drug penetration into ocular tissues. Compared to the number of available antibiotics, the number of available antifungal drugs is very modest. In addition, the few effective drugs are difficult to obtain in LMICs with the highest numbers of cases, as well as in many developed countries with lower incidences. Although *Fusarium* keratitis is a serious burden in some regions of the world, there are still no clear treatment guidelines available.

Based on available evidence, it is advisable to use topical 5% NAT as a first line treatment. In countries where NAT is not available, the choice of first-line therapy is difficult given the lack of clear evidence regarding treatment efficacy. Options include 0.15% AMB, 1% VCZ, 0.2% CHX, or 2% econazole. Decisions should be based on local fungal susceptibility patterns (if known), drug availability (including the ability to manufacture “in house” as with AMB or CHX), and cost. Some of these agents, such as AMB and CHX, may be useful as adjunctive topical agents; however, more research is required to explore this further. In recalcitrant cases, intrastromal and intracameral applications of AMB and VCZ can be considered as a way to increase drug bioavailability. However, available evidence is poor. We suspect that melting of the lens, which occurred in one of our *Fusarium* keratitis cases (Figure 3), might have been due to toxic effects of the drugs repeatedly injected into the anterior chamber, although we have found no publications describing such adverse effect. TPK or TLK are treatment options for cases not responding to medical treatments. Based on actual research, to lower the rate of recurrence and graft failure, this surgical treatment should not be delayed. Promising treatment options include photodynamic therapy with the use of rose bengal and green light, but, for now, this requires further research.

In our clinical cases presented in the figure collage, we described two extremely different courses of disease and treatment. In one case, a rapid microbiological diagnosis enabled early introduction of the appropriate treatment. At the time, when the treatment started the infiltrate was small and did not penetrate deep into cornea. The patient had excellent visual acuity. Compared to the most common *F. solani SC*, *F. oxysporum SC* has a better susceptibility profile, and the treatment with topical VCZ has proved sufficient.

In the second case, given the patient’s reluctance to be treated promptly, the patient likely presented too late to our department for any treatment to be effective. It is unclear whether it was primary *Fusarium* keratitis or if *Fusarium* infection developed secondary to treatment delay, and a prolonged course of topical steroids, in the presence of a large epithelial defect. At the presentation, the infiltrate was large and deep and was accompanied by hypopyon, meaning that the patient had three risk factors of recurrence at the time of TPK. In addition, for procedural reasons, we could not implement the proper antifungal treatment at the time of suspicion of the fungal infection. In this case, we were unable to determine the *Fusarium* subspecies, which made the treatment even more difficult. It is doubtful if an early second keratoplasty could have changed the course of this fulminant infection.

In conclusion, because *Fusarium* keratitis poses a serious threat for vision and, in severe cases, even the eye, with limited treatment options—prompt diagnosis and early introduction of antifungal treatment are essential. Improving the availability of the few effective drugs in LMICs with the highest incidence rates is an urgent issue. There is still a lot to be learned about medical and surgical treatments of this ocular emergency. The development of new antifungals or new routes of administration may bring about breakthroughs in the treatment of this extremely challenging infection.

## Figures and Tables

**Table 1:** Dosages and methods of administration of the most commonly used antifungals.

Antifungal	Topical	Intrastromal	Intracameral	Oral	References
Natamycin	5%, hourly in first 48 h	10 µg/0.1 mL	not used	not used	[81,87]
Amphotericin B	0.15%, rarely used	5 µg/0.1 mL, can be repeated with 72 h interval	5 µg/0.1 mL, can be repeated	not used	[81,82,83,84]
Voriconazole	1%, hourly in first 48 h	50 µg/0.1 mL, can be repeated with 72 h interval	50 µg/0.1 mL, 100 µg/0.1 mL, can be repeated	2 × 400 mg in first 24 h, 2 × 200 mg in the next days	[70,79,81,85,86,87]

## Data Availability

Not applicable.

## Supplementary Materials

The following are available online at https://www.mdpi.com/article/10.3390/jcm10235468/s1, Search strategy overview.

## References

[1] Brown L., Leck A.K., Gichangi M., Burton M.J., Denning D.W. (2020). The global incidence and diagnosis of fungal keratitis. Lancet Infect. Dis..

[2] Hoffman J., Burton M., Leck A. (2021). Mycotic Keratitis—A Global Threat from the Filamentous Fungi. J. Fungi.

[3] Thomas P.A., Leck A.K., Myatt M. (2005). Characteristic clinical features as an aid to the diagnosis of suppurative keratitis caused by filamentous fungi. Br. J. Ophthalmol..

[4] Sun Y., Chandra J., Mukherjee P., Szczotka-Flynn L., Ghannoum M.A., Pearlman E. (2010). A Murine Model of Contact Lens–Associated *Fusarium* Keratitis. Investig. Opthalmol. Vis. Sci..

[5] Calvillo-Medina R.P., Reyes J.P., Barba-Escoto L., Bautista-Hernandez L.A., Campos-Guillén J., Jones G.H., de Lucio V.M.B. (2019). Proteome analysis of biofilm produced by a *Fusarium* falciforme keratitis infectious agent. Microb. Pathog..

[6] Mukherjee P.K., Chandra J., Yu C., Sun Y., Pearlman E., Ghannoum M.A. (2012). Characterization of *Fusarium* Keratitis Outbreak Isolates: Contribution of Biofilms to Antimicrobial Resistance and Pathogenesis. Investig. Opthalmol. Vis. Sci..

[7] Ponce-Angulo D.G., Bautista-Hernández L.A., Calvillo-Medina R.P., Castro-Tecorral F.I., Aparicio-Ozores G., Villegas E.O.L., Ribas-Aparicio R.M., de Lucio V.M.B. (2020). Microscopic characterization of biofilm in mixed keratitis in a novel murine model. Microb. Pathog..

[8] Zhang X., Sun X., Wang Z., Zhang Y., Hou W. (2012). Keratitis-Associated Fungi Form Biofilms with Reduced Antifungal Drug Susceptibility. Investig. Opthalmol. Vis. Sci..

[9] Córdova-Alcántara I.M., Venegas-Cortés D.L., Martínez-Rivera M., Pérez N.O., Rodriguez-Tovar A.V. (2019). Biofilm characterization of *Fusarium* solani keratitis isolate: Increased resistance to antifungals and UV light. J. Microbiol..

[10] Madhu S., Gajjar D., Sharma S. (2020). Identification of Proteases: Carboxypeptidase and Aminopeptidase as Putative Virulence Factors of *Fusarium* solani Species Complex. Open Microbiol. J..

[11] Naiker S., Odhav B. (2004). Mycotic keratitis: Profile of *Fusarium* species and their mycotoxins. Mykotische Keratitis: Profil von *Fusarium*-Arten und ihren Mykotoxinen. Mycoses.

[12] Hu J., Hu Y., Chen S., Dong C., Zhang J., Li Y., Yang J., Han X., Zhu X., Xu G. (2014). Role of activated macrophages in experimental *Fusarium* solani keratitis. Exp. Eye Res..

[13] Karthikeyan R.S.G., Leal S., Prajna N.V., Dharmalingam K., Geiser D.M., Pearlman E., Lalitha P. (2011). Expression of Innate and Adaptive Immune Mediators in Human Corneal Tissue Infected with *Aspergillus* or *Fusarium*. J. Infect. Dis..

[14] Li Q., Gao X.-R., Cui H.-P., Lang L.-L., Xie X.-W., Chen Q. (2016). Time-dependent matrix metalloproteinases and tissue inhibitor of metalloproteinases expression change in *Fusarium solani* keratitis. Int. J. Ophthalmol..

[15] Mitchell B.M., Wu T.G., Chong E.-M., Pate J.C., Wilhelmus K.R. (2007). Expression of Matrix Metalloproteinases 2 and 9 in Experimental Corneal Injury and Fungal Keratitis. Cornea.

[16] Cong L., Xia Y.-P., Zhao G.-Q., Lin J., Xu Q., Hu L.-T., Qu J.-Q., Peng X.-D. (2015). Expression of vitamin D receptor and cathelicidin in human corneal epithelium cells during *Fusarium solani* infection. Int. J. Ophthalmol..

[17] Jin X., Qin Q., Tu L., Zhou X., Lin Y., Qu J. (2007). Toll-like receptors (TLRs) expression and function in response to inactivate hyphae of *Fusarium* solani in immortalized human corneal epithelial cells. Mol. Vis..

[18] Ananthi S., Prajna N.V., Lalitha P., Valarnila M., Dharmalingam K. (2013). Pathogen Induced Changes in the Protein Profile of Human Tears from *Fusarium* Keratitis Patients. PLoS ONE.

[19] Chidambaram J.D., Prajna N.V., Srikanthi P., Lanjewar S., Shah M., Elakkiya S., Lalitha P., Burton M.J. (2018). Epidemiology, risk factors, and clinical outcomes in severe microbial keratitis in South India. Ophthalmic Epidemiol..

[20] E Oldenburg C., Prajna V.N., Prajna L., Krishnan T., Mascarenhas J., Vaitilingam C.M., Srinivasan M., See C.W., Cevallos V., E Zegans M. (2011). Clinical signs in dematiaceous and hyaline fungal keratitis. Br. J. Ophthalmol..

[21] Sharma S. (2011). Diagnosis of infectious diseases of the eye. Eye.

[22] Chidambaram J.D., Prajna N.V., Larke N.L., Palepu S., Lanjewar S., Shah M., Elakkiya S., Lalitha P., Carnt N., Vesaluoma M.H. (2016). Prospective Study of the Diagnostic Accuracy of the In Vivo Laser Scanning Confocal Microscope for Severe Microbial Keratitis. Ophthalmology.

[23] da Rosa P.D., Nunes A., Borges R., Batista B., Fuentefria A.M., Goldani L. (2018). In vitro susceptibility and multilocus sequence typing of *Fusarium* isolates causing keratitis. J. Mycol. Med..

[24] Azor M., Gené J., Cano J., Guarro J. (2007). Universal In Vitro Antifungal Resistance of Genetic Clades of the *Fusarium* solani Species Complex. Antimicrob. Agents Chemother..

[25] Manikandan P., Abdel-Hadi A., Randhir Babu Singh Y., Revathi R., Anita R., Banawas S., Bin Dukhyil A.A., Alshehri B., Shobana C.S., Panneer Selvam K. (2019). Fungal Keratitis: Epidemiology, Rapid Detection, and Antifungal Susceptibilities of *Fusarium* and *Aspergillus* Isolates from Corneal Scrapings. BioMed Res. Int..

[26] Liang Q.-F., Jin X.-Y., Wang X.-L., Sun X.-G. (2009). Effect of topical application of terbinafine on fungal keratitis. Chin. Med. J..

[27] Xie L., Zhai H., Zhao J., Sun S., Shi W., Dong X. (2008). Antifungal Susceptibility for Common Pathogens of Fungal Keratitis in Shandong Province, China. Am. J. Ophthalmol..

[28] Lemke A., Kiderlen A.F., Kayser O. (2005). Amphotericin B. Appl. Microbiol. Biotechnol..

[29] Te Welscher Y.M., Napel H.H.T., Balagué M.M., Souza C.M., Riezman H., de Kruijff B., Breukink E. (2008). Natamycin Blocks Fungal Growth by Binding Specifically to Ergosterol without Permeabilizing the Membrane. J. Biol. Chem..

[30] Te Welscher Y.M., van Leeuwen M.R., de Kruijff B., Dijksterhuis J., Breukink E. (2012). Polyene antibiotic that inhibits membrane transport proteins. Proc. Natl. Acad. Sci. USA.

[31] Sanati H., Belanger P., Fratti R., Ghannoum M. (1997). A new triazole, voriconazole (UK-109,496), blocks sterol biosynthesis in Candida albicans and Candida krusei. Antimicrob. Agents Chemother..

[32] O’Day D.M., Head W.S., Robinson R.D., Clanton J.A. (1986). Corneal penetration of topical amphotericin B and natamycin. Curr. Eye Res..

[33] Müller G.G., Kara-José N., de Castro R.S. (2013). Antifungals in eye infections: Drugs and routes of administration. Rev. Bras. Oftalmol..

[34] Patil A., Lakhani P., Majumdar S. (2017). Current perspectives on natamycin in ocular fungal infections. J. Drug Deliv. Sci. Technol..

[35] Qiu S., Zhao G.-Q., Lin J., Wang X., Hu L.-T., Du Z.-D., Wang Q., Zhu C.-C. (2015). Natamycin in the treatment of fungal keratitis: A systematic review and Meta-analysis. Int. J. Ophthalmol..

[36] Wang L., Wang L., Han L., Yin W. (2015). Study of Pathogens of Fungal Keratitis and the Sensitivity of Pathogenic Fungi to Therapeutic Agents with the Disk Diffusion Method. Curr. Eye Res..

[37] Sharma S., Sahu S.K., Garg P., Pradhan L., Nalamada S., Das S. (2011). Natamycin in the treatment of keratomycosis: Correlation of treatment outcome and in vitro susceptibility of fungal isolates. Indian J. Ophthalmol..

[38] Prajna N.V., Krishnan T., Mascarenhas J., Rajaraman R., Prajna L., Srinivasan M., Raghavan A., Oldenburg C.E., Ray K.J., Zegans M.E. (2013). The Mycotic Ulcer Treatment Trial: A randomized trial comparing natamycin vs. voriconazole. JAMA Ophthalmol..

[39] FlorCruz N.V., Evans J.R. (2015). Medical interventions for fungal keratitis. Cochrane Database Syst. Rev..

[40] Walther G., Stasch S., Kaerger K., Hamprecht A., Roth M., Cornely O.A., Geerling G., MacKenzie C., Kurzai O., von Lilienfeld-Toal M. (2017). *Fusarium* Keratitis in Germany. J. Clin. Microbiol..

[41] Burton M.J., Pithuwa J., Okello E., Afwamba I., Onyango J.J., Oates F., Chevallier C., Hall A.B. (2011). Microbial Keratitis in East Africa: Why are the Outcomes so Poor?. Ophthalmic Epidemiol..

[42] (2021). WHO Model List of Essential Medicines—22nd List. https://www.who.int/publications-detail-redirect/WHO-MHP-HPS-EML-2021.02.

[43] Kaur I.P., Kakkar S. (2010). Topical delivery of antifungal agents. Expert Opin. Drug Deliv..

[44] Oechsler R.A., Yamanaka T.M., Bispo P.J., Sartori J., Yu M.C.Z., A Melo A.S., Miller D., Hofling-Lima A.L. (2013). *Fusarium* keratitis in Brazil: Genotyping, in vitro susceptibilities, and clinical outcomes. Clin. Ophthalmol..

[45] Gajjar D.U., Pal A.K., Ghodadra B.K., Vasavada A.R. (2013). Microscopic Evaluation, Molecular Identification, Antifungal Susceptibility, and Clinical Outcomes in *Fusarium*, *Aspergillus* and, Dematiaceous Keratitis. BioMed Res. Int..

[46] Kawakami H., Inuzuka H., Hori N., Takahashi N., Ishida K., Mochizuki K., Ohkusu K., Muraosa Y., Watanabe A., Kamei K. (2015). Inhibitory effects of antimicrobial agents against *Fusarium* species. Med. Mycol..

[47] Lalitha P., Shapiro B.L., Srinivasan M., Prajna N.V., Acharya N.R., Fothergill A.W., Ruiz J., Chidambaram J., Maxey K.J., Hong K.C. (2007). Antimicrobial Susceptibility of *Fusarium*, *Aspergillus*, and Other Filamentous Fungi Isolated from Keratitis. Arch. Ophthalmol..

[48] Prajna N.V., Radhakrishnan N., Lalitha P., Austin A., Ray K.J., Keenan J.D., Porco T.C., Lietman T.M., Rose-Nussbaumer J. (2020). Cross-Linking–Assisted Infection Reduction: A Randomized Clinical Trial Evaluating the Effect of Adjuvant Cross-Linking on Outcomes in Fungal Keratitis. Ophthalmology.

[49] Kimakura M., Usui T., Yokoo S., Nakagawa S., Yamagami S., Amano S. (2014). Toxicity of Topical Antifungal Agents to Stratified Human Cultivated Corneal Epithelial Sheets. J. Ocul. Pharmacol. Ther..

[50] Foster C.S., Lass J.H., Moran-Wallace K., Giovanoni R. (1981). Ocular Toxicity of Topical Antifungal Agents. Arch. Ophthalmol..

[51] Dos Santos C.O., Kolwijck E., Van Rooij J., Stoutenbeek R., Visser N., Cheng Y.Y., Santana N.T.Y., Verweij P.E., Eggink C.A. (2020). Epidemiology and Clinical Management of *Fusarium* keratitis in the Netherlands, 2005–2016. Front. Cell. Infect. Microbiol..

[52] Zhao X., Tong Y., Wang X., Zhang X., Chen S., Lu H. (2018). Comparison of the Ocular Penetration and Pharmacokinetics Between Natamycin and Voriconazole After Topical Instillation in Rabbits. J. Ocul. Pharmacol. Ther..

[53] Vorwerk C.K., Streit F., Binder L., Tuchen S., Knop C., Behrens-Baumann W. (2008). Aqueous humor concentration of voriconazole after topical administration in rabbits. Graefe’s Arch. Clin. Exp. Ophthalmol..

[54] Prajna V.N., Lalitha P.S., Mascarenhas J., Krishnan T., Srinivasan M., Vaitilingam C.M., E Oldenburg C., Sy A., Keenan J.D., Porco T.C. (2012). Natamycin and voriconazole in *Fusarium* and *Aspergillus* keratitis: Subgroup analysis of a randomised controlled trial: Table 1. Br. J. Ophthalmol..

[55] Lalitha P., Vijaykumar R., Prajna N.V., Fothergill A.W. (2008). In Vitro Natamycin Susceptibility of Ocular Isolates of *Fusarium* and *Aspergillus* Species: Comparison of Commercially Formulated Natamycin Eye Drops to Pharmaceutical-Grade Powder. J. Clin. Microbiol..

[56] Ray K.J., Lalitha P., Prajna N.V., Rajaraman R., Krishnan T., Srinivasan M., Ryg P., McLeod S., Acharya N.R., Lietman T.M. (2017). The Utility of Repeat Culture in Fungal Corneal Ulcer Management: A Secondary Analysis of the MUTT-I Randomized Clinical Trial. Am. J. Ophthalmol..

[57] Ray K.J., Prajna N.V., Lalitha P., Rajaraman R., Krishnan T., Patel S., Das M., Shah R., Dhakhwa K., McLeod S.D. (2018). The Significance of Repeat Cultures in the Treatment of Severe Fungal Keratitis. Am. J. Ophthalmol..

[58] Arora R., Goyal J., Gupta D., Kaur R. (2011). Voriconazole versus natamycin as primary treatment in fungal corneal ulcers. Clin. Exp. Ophthalmol..

[59] Prajna N.V., Prajna N.V., Mascarenhas J., Krishnan T., Reddy P.R., Prajna L., Srinivasan M., Vaitilingam C.M., Hong K.C., Lee A.M. (2010). Comparison of Natamycin and Voriconazole for the Treatment of Fungal Keratitis. Arch. Ophthalmol..

[60] Sharma S., Das S., Virdi A., Fernandes M., Sahu S.K., Kumar Koday N., Ali M.H., Garg P., Motukupally S.R. (2015). Re-appraisal of topical 1% voriconazole and 5% natamycin in the treatment of fungal keratitis in a randomised trial. Br. J. Ophthalmol..

[61] Oechsler R.A., Feilmeier M.R., Miller D., Shi W., Hofling-Lima A.L., Alfonso E.C. (2013). *Fusarium* Keratitis: Genotyping, in vitro susceptibility and clinical outcomes. Cornea.

[62] Aung T.T., Chor W.H.J., Lynn M.N., Chan A.S.Y., Tan D.T., Beuerman R.W. (2020). Rabbit Fungal Keratitis Model of *Fusarium* solani Tested Against Three Commercially Available Antifungal Drugs. Eye Contact.

[63] Mahashabde S., Nahata M.C., Shrivastava U. (1987). A comparative study of anti-fungal drugs in mycotic corneal ulcer. Indian J. Ophthalmol..

[64] Arora I., Kulshrestha O.P., Upadhaya S. (1983). Treatment of fungal corneal ulcers with econazole. Indian J. Ophthalmol..

[65] Prajna N.V., John R.K., Nirmalan P.K., Lalitha P., Srinivasan M. (2003). A randomised clinical trial comparing 2% econazole and 5% natamycin for the treatment of fungal keratitis. Br. J. Ophthalmol..

[66] Lalitha P., Shapiro B.L., Loh A.R., Fothergill A.W., Prajna N.V., Srinivasan M., Oldenburg C.E., Quigley D.A., Chidambaram J.D., McLeod S.D. (2011). Amphotericin B and natamycin are not synergistic in vitro against *Fusarium* and *Aspergillus* spp. isolated from keratitis. Br. J. Ophthalmol..

[67] Sradhanjali S., Yein B., Sharma S., Das S. (2018). In vitro synergy of natamycin and voriconazole against clinical isolates of *Fusarium*, Candida, *Aspergillus* and Curvularia spp. Br. J. Ophthalmol..

[68] Al-Hatmi A.M.S., Meletiadis J., Curfs-Breuker I., Bonifaz A., Meis J.F., De Hoog G.S. (2016). In vitro combinations of natamycin with voriconazole, itraconazole and micafungin against clinical *Fusarium* strains causing keratitis. J. Antimicrob. Chemother..

[69] He Y., Zhou L., Gao C., Han L., Xu Y. (2017). Rifampin Enhances the Activity of Amphotericin B against *Fusarium* solani Species Complex and *Aspergillus* flavus Species Complex Isolates from Keratitis Patients. Antimicrob. Agents Chemother..

[70] Prajna N.V., Krishnan T., Rajaraman R., Patel S., Srinivasan M., Das M., Ray K.J., O’Brien K.S., Oldenburg C.E., McLeod S.D. (2016). Effect of Oral Voriconazole on Fungal Keratitis in the Mycotic Ulcer Treatment Trial II (MUTT II): A Randomized Clinical Trial. JAMA Ophthalmol..

[71] Prajna N.V., Krishnan T., Rajaraman R., Patel S., Shah R., Srinivasan M., Devi L., Das M., Ray K.J., O’Brien K.S. (2017). Adjunctive Oral Voriconazole Treatment of *Fusarium* Keratitis: A Secondary Analysis From the Mycotic Ulcer Treatment Trial II. JAMA Ophthalmol..

[72] Sharma N., Singhal D., Maharana P.K., Sinha R., Agarwal T., Upadhyay A.D., Velpandian T., Satpathy G., Titiyal J.S. (2017). Comparison of Oral Voriconazole Versus Oral Ketoconazole as an Adjunct to Topical Natamycin in Severe Fungal Keratitis: A Randomized Controlled Trial. Cornea.

[73] Ravuri S., Mittal R., Bagga B. (2015). Topical 5% Natamycin with Oral Ketoconazole in Filamentous Fungal Keratitis: A Randomized Controlled Trial. Asia-Pac. J. Ophthalmol..

[74] Nejabat M., Yaqubi N., Khosravi A., Zomorodian K., Ashraf M.J., Salouti R. (2016). Therapeutic Effect of Intrastromal Voriconazole, Topical Voriconazole, and Topical Natamycin on *Fusarium* Keratitis in Rabbit. J. Ophthalmol..

[75] Kalaiselvi G., Narayana S., Krishnan T., Sengupta S. (2015). Intrastromal voriconazole for deep recalcitrant fungal keratitis: A case series. Br. J. Ophthalmol..

[76] Siatiri H., Daneshgar F., Siatiri N., Khodabande A. (2011). The Effects of Intrastromal Voriconazole Injection and Topical Voriconazole in the Treatment of Recalcitrant *Fusarium* Keratitis. Cornea.

[77] Sharma N., Agarwal P., Sinha R., Titiyal J.S., Velpandian T., Vajpayee R.B. (2011). Evaluation of intrastromal voriconazole injection in recalcitrant deep fungal keratitis: Case series. Br. J. Ophthalmol..

[78] Eguchi H., Hayashi Y., Miyamoto T., Hotta F., Mitamura Y., Niki M. (2014). Ineffectiveness of intrastromal voriconazole for filamentous fungal keratitis. Clin. Ophthalmol..

[79] Narayana S., Krishnan T., Ramakrishnan S., Samantaray P.P., Austin A., Pickel J., Porco T., Lietman T., Rose-Nussbaumer J. (2019). Mycotic Antimicrobial Localized Injection: A Randomized Clinical Trial Evaluating Intrastromal Injection of Voriconazole. Ophthalmology.

[80] Sharma N., Chacko J., Velpandian T., Titiyal J.S., Sinha R., Satpathy G., Tandon R., Vajpayee R.B. (2013). Comparative Evaluation of Topical versus Intrastromal Voriconazole as an Adjunct to Natamycin in Recalcitrant Fungal Keratitis. Ophthalmology.

[81] Saluja G., Sharma N., Agarwal R., Sharma H.P., Singhal D., Kumar Maharana P., Sinha R., Agarwal T., Velpandian T., Titiyal J.S. (2021). Comparison of Safety and Efficacy of Intrastromal Injections of Voriconazole, Amphotericin B and Natamycin in Cases of Recalcitrant Fungal Keratitis: A Randomized Controlled Trial. Clin. Ophthalmol..

[82] Yilmaz S., Ture M., Maden A. (2007). Efficacy of Intracameral Amphotericin B Injection in the Management of Refractory Keratomycosis and Endophthalmitis. Cornea.

[83] Hu J., Zhang J., Li Y., Han X., Zheng W., Yang J., Xu G. (2016). A Combination of Intrastromal and Intracameral Injections of Amphotericin B in the Treatment of Severe Fungal Keratitis. J. Ophthalmol..

[84] Sharma N., Sankaran P., Agarwal T., Arora T., Chawla B., Titiyal J.S., Tandon R., Satapathy G., Vajpayee R.B. (2016). Evaluation of Intracameral Amphotericin B in the Management of Fungal Keratitis: Randomized Controlled Trial. Ocul. Immunol. Inflamm..

[85] Killani S.P., Atti S., Gupta A., Pujala S.R., Goli S., Mahendra S., Adapala A., Budamakayala K. (2015). Intracameral and Intracorneal Voriconazole in Deep Keratomycosis with Endothelial Plaque. J. Evol. Med. Dent. Sci..

[86] Shen Y.-C., Wang C.-Y., Tsai H.-Y., Lee H.-N. (2010). Intracameral Voriconazole Injection in the Treatment of Fungal Endophthalmitis Resulting from Keratitis. Am. J. Ophthalmol..

[87] Sharma N., Sahay P., Maharana P.K., Singhal D., Saluja G., Bandivadekar P., Chako J., Agarwal T., Sinha R., Titiyal J.S. (2019). Management Algorithm for Fungal Keratitis: The TST (Topical, Systemic, and Targeted Therapy) Protocol. Cornea.

[88] Sponsel W.E., Graybill J.R., Nevarez H.L., Dang D. (2002). Ocular and systemic posaconazole (SCH-56592) treatment of invasive *Fusarium solani* keratitis and endophthalmitis. Br. J. Ophthalmol..

[89] Tu E.Y., McCartney D.L., Beatty R.F., Springer K.L., Levy J., Edward D. (2007). Successful Treatment of Resistant Ocular Fusariosis With Posaconazole (SCH-56592). Am. J. Ophthalmol..

[90] Altun A., Kurna S.A., Sengor T., Altun G., Olcaysu O.O., Aki S.F., Simsek M.H. (2014). Effectiveness of Posaconazole in Recalcitrant Fungal Keratitis Resistant to Conventional Antifungal Drugs. Case Rep. Ophthalmol. Med..

[91] Soleimani M., Salehi Z., Fattahi A., Lotfali E., Yassin Z., Ghasemi R., Abedinifar Z., Kouhsari E., Ahmadkhani F., Mirkalantari S. (2020). Ocular Fungi: Molecular Identification and Antifungal Susceptibility Pattern to Azoles. Jundishapur J. Microbiol..

[92] Todokoro D., Suzuki T., Tamura T., Makimura K., Yamaguchi H., Inagaki K., Akiyama H. (2019). Efficacy of Luliconazole Against Broad-Range Filamentous Fungi Including *Fusarium* solani Species Complex Causing Fungal Keratitis. Cornea.

[93] Neoh C.F., Daniell M., Chen S.C.-A., Stewart K., Kong D.C.M. (2014). Clinical utility of caspofungin eye drops in fungal keratitis. Int. J. Antimicrob. Agents.

[94] Li J., Li Z., Liang Z., Han L., Feng H., He S., Zhang J. (2018). Fabrication of a drug delivery system that enhances antifungal drug corneal penetration. Drug Deliv..

[95] Wang F., Zhao L., Song F., Wu J., Zhou Q., Xie L. (2021). Hybrid natural hydrogels integrated with voriconazole-loaded microspheres for ocular antifungal applications. J. Mater. Chem. B.

[96] Jain A., Shah S.G., Chugh A. (2015). Cell Penetrating Peptides as Efficient Nanocarriers for Delivery of Antifungal Compound, Natamycin for the Treatment of Fungal Keratitis. Pharm. Res..

[97] Rohira H., Shankar S., Yadav S., Shah S.G., Chugh A. (2021). Enhanced in vivo antifungal activity of novel cell penetrating peptide natamycin conjugate for efficient fungal keratitis management. Int. J. Pharm..

[98] Jansook P., Maw P.D., Soe H.M.S.H., Chuangchunsong R., Saiborisuth K., Payonitikarn N., Autthateinchai R., Pruksakorn P. (2020). Development of amphotericin B nanosuspensions for fungal keratitis therapy: Effect of self-assembled γ-cyclodextrin. J. Pharm. Investig..

[99] Rahman M.R., Minassian D.C., Srinivasan M., Martin M.J., Johnson G.J. (1997). Trial of chlorhexidine gluconate for fungal corneal ulcers. Ophthalmic Epidemiol..

[100] Rahman M.R., Johnson G.J., Husain R., Howlader S.A., Minassian D.C. (1998). Randomised trial of 0.2% chlorhexidine gluconate and 2.5% natamycin for fungal keratitis in Bangladesh. Br. J. Ophthalmol..

[101] Dos Santos C.O., Kolwijck E., van der Lee H.A., Tehupeiory-Kooreman M.C., Al-Hatmi A.M.S., Matayan E., Burton M.J., Eggink C.A., Verweij P. (2019). In Vitro Activity of Chlorhexidine Compared with Seven Antifungal Agents against 98 *Fusarium* Isolates Recovered from Fungal Keratitis Patients. Antimicrob. Agents Chemother..

[102] Dos Santos C.O., Hanemaaijer N., Ye J., van der Lee H., Verweij P., Eggink C. (2021). Chlorhexidine for the Treatment of *Fusarium* Keratitis: A Case Series and Mini Review. J. Fungi.

[103] Jiang T., Tang J., Wu Z., Sun Y., Tan J., Yang L. (2020). The combined utilization of Chlorhexidine and Voriconazole or Natamycin to combat *Fusarium* infections. BMC Microbiol..

[104] Arunga S., Mbarak T., Ebong A., Mwesigye J., Kuguminkiriza D., Mohamed A., Hoffman J.J., Leck A., Hu V., Burton M. (2021). Chlorhexidine gluconate 0.2% as a treatment for recalcitrant fungal keratitis in Uganda: A pilot study. BMJ Open Ophthalmol..

[105] Fernández-Ferreiro A., Santiago-Varela M., Gil-Martínez M., González-Barcia M., Luaces-Rodríguez A., Díaz-Tome V., Pardo M., Méndez J.B., Piñeiro-Ces A., Rodríguez-Ares M.T. (2017). In Vitro Evaluation of the Ophthalmic Toxicity Profile of Chlorhexidine and Propamidine Isethionate Eye Drops. J. Ocul. Pharmacol. Ther..

[106] Steinsapir K.D., Woodward J.A. (2017). Chlorhexidine Keratitis: Safety of Chlorhexidine as a Facial Antiseptic. Dermatol. Surg..

[107] Hamed L.M., Ellis F.D., Boudreault G., Ii F.M.W., Helveston E.M. (1987). Hibiclens Keratitis. Am. J. Ophthalmol..

[108] Merani R., McPherson Z.E., Luckie A.P., Gilhotra J.S., Runciman J., Durkin S., Muecke J., Donaldson M., Aralar A., Rao A. (2016). Aqueous Chlorhexidine for Intravitreal Injection Antisepsis: A Case Series and Review of the Literature. Ophthalmology.

[109] Ali F.S., Jenkins T.L., Boparai R.S., Obeid A., Ryan M.E., Wibblesman T.D., Chiang A., Garg S.J., Levin H.J., Xu D. (2021). Aqueous Chlorhexidine Compared with Povidone-Iodine as Ocular Antisepsis before Intravitreal Injection: A Randomized Clinical Trial. Ophthalmol. Retin..

[110] Murthy S., Hawksworth N.R., Cree I. (2002). Progressive Ulcerative Keratitis Related to the Use of Topical Chlorhexidine Gluconate (0.02%). Cornea.

[111] Okuda T., Funasaka M., Arimitsu M., Umeda T., Wakita K., Koga Y. (1994). Anaphylactic shock by ophthalmic wash solution containing chlorhexidine. Masui.

[112] Hoffman J.J., Yadav R., Das Sanyam S., Chaudhary P., Roshan A., Singh S.K., Arunga S., Matayan E., MacLeod D., Weiss H.A. (2020). Topical chlorhexidine 0.2% versus topical natamycin 5% for fungal keratitis in Nepal: Rationale and design of a randomised controlled non-inferiority trial. BMJ Open.

[113] ISRCTN—ISRCTN87195453: A Comparison of Two Treatment Regimes for the Treatment of Fungal Eye Infections in East Africa. https://www.isrctn.com/ISRCTN87195453?q=chlorhexidine%20natamycin&filters=&sort=&offset=1&totalResults=2&page=1&pageSize=10&searchType=basic-search.

[114] Diongue K., Sow A., Nguer M., Seck M., Ndiaye M., Badiane A., Ndoye N., Diallo M., Diop A., Ndiaye Y. (2015). Kératomycose à *Fusarium* oxysporum traitée par l’association de la povidone iodée en collyre et du fluconazole per os. J. Mycol. Méd..

[115] De Oliveira L.A., Takata T.T., Shiguematsu A., Júnior L.A.S.M., Gompertz O.F., De Sousa L.B., Mannis M.J. (2008). Effect of topical 0.5% povidone-iodine compared to 5% natamycin in fungal keratitis caused by *Fusarium* solani in a rabbit model: A pilot study. Arq. Bras. Oftalmol..

[116] Martin M.J., Rahman M.R., Johnson G.J., Srinivasan M., Clayton Y.M. (1995). Mycotic keratitis: Susceptibility to antiseptic agents. Int. Ophthalmol..

[117] Panda A., Ahuja R., Biswas N.R., Satpathy G., Khokhar S. (2003). Role of 0.02% Polyhexamethylene Biguanide and 1% Povidone Iodine in Experimental *Aspergillus* Keratitis. Cornea.

[118] Behrens-Baumann W., Seibold M., Hofmüller W., Walter S., Haeberle H., Wecke T., Tammer I., Tintelnot K. (2012). Benefit of Polyhexamethylene Biguanide in *Fusarium* Keratitis. Ophthalmic Res..

[119] Gulias-Cañizo R., Benatti A., De Wit-Carter G., Hernández-Quintela E., Sánchez-Huerta V. (2020). Photoactivated Chromophore for Keratitis-Corneal Collagen Cross-Linking (PACK-CXL) Improves Outcomes of Treatment-Resistant Infectious Keratitis. Clin. Ophthalmol..

[120] Said D.G., Elalfy M.S., Gatzioufas Z., El-Zakzouk E.S., Hassan M.A., Saif M.Y., Zaki A.A., Dua H.S., Hafezi F. (2014). Collagen Cross-Linking with Photoactivated Riboflavin (PACK-CXL) for the Treatment of Advanced Infectious Keratitis with Corneal Melting. Ophthalmology.

[121] Tabibian D., Mazzotta C., Hafezi F. (2016). PACK-CXL: Corneal cross-linking in infectious keratitis. Eye Vis..

[122] AlShehri J.M., Caballero-Lima D., Hillarby M.C., Shawcross S.G., Brahma A., Carley F., Read N.D., Radhakrishnan H. (2016). Evaluation of Corneal Cross-Linking for Treatment of Fungal Keratitis: Using Confocal Laser Scanning Microscopy on an Ex Vivo Human Corneal Model. Investig. Opthalmol. Vis. Sci..

[123] Zhu Z., Zhang H., Yue J., Liu S., Li Z., Wang L. (2018). Antimicrobial efficacy of corneal cross-linking in vitro and in vivo for *Fusarium* solani: A potential new treatment for fungal keratitis. BMC Ophthalmol..

[124] Kalkanci A., Bilgihan K., Ozdemir H.B., Saglam A.S.Y., Karakurt F., Erdogan M. (2018). Corneal Cross-Linking Has No Effect on Matrix Metalloproteinase 9 and 13 Levels During Fungal Keratitis on the Early Stage. Mycopathologia.

[125] Sun B., Li Z.-W., Yu H.-Q., Tao X.-C., Zhang Y., Mu G.-Y. (2014). Evaluation of the in vitro antimicrobial properties of ultraviolet A/riboflavin mediated crosslinking on Candida albicans and *Fusarium solani*. Int. J. Ophthalmol..

[126] Kashiwabuchi R.T., Carvalho F.R.S., Khan Y.A., Hirai F., Campos M.S., McDonnell P.J. (2013). Assessment of fungal viability after long-wave ultraviolet light irradiation combined with riboflavin administration. Graefe Arch. Clin. Exp. Ophthalmol..

[127] Uddaraju M., Mascarenhas J., Das M.R., Radhakrishnan N., Keenan J.D., Prajna L., Prajna V.N. (2015). Corneal Cross-linking as an Adjuvant Therapy in the Management of Recalcitrant Deep Stromal Fungal Keratitis: A Randomized Trial. Am. J. Ophthalmol..

[128] Ting D.S.J., Henein C., Said D.G., Dua H.S. (2020). Re: Prajna et al.: Cross-Linking-Assisted Infection Reduction (CLAIR): A randomized clinical trial evaluating the effect of adjuvant cross-linking on outcomes in fungal keratitis (*Ophthalmology*. 2020;127:159–166). Ophthalmology.

[129] Hafezi F., Torres-Netto E., Hillen M.J. (2020). Re: Prajna et al.: Cross-Linking—Assisted Infection Reduction: A randomized clinical trial evaluating the effect of adjuvant cross-linking on outcomes in fungal keratitis (Ophthalmology. 2020;127:159–166). Ophthalmology.

[130] Wei A., Wang K., Wang Y., Gong L., Xu J., Shao T. (2019). Evaluation of corneal cross-linking as adjuvant therapy for the management of fungal keratitis. Graefe Arch. Clin. Exp. Ophthalmol..

[131] Balparda K., Mejia-Turizo J.C., Herrera-Chalarca T. (2017). Simultaneous Noncentered Photoactivated Chromophore for Keratitis-Corneal Collagen Cross-Linking and Penetrating Keratoplasty for Treatment of Severe Marginal *Fusarium* spp. Keratitis: A Description of a New Surgical Technique. Case Rep. Ophthalmol. Med..

[132] Arboleda A., Miller D., Cabot F., Taneja M., Aguilar M.C., Alawa K., Amescua G., Yoo S.H., Parel J.-M. (2014). Assessment of Rose Bengal Versus Riboflavin Photodynamic Therapy for Inhibition of Fungal Keratitis Isolates. Am. J. Ophthalmol..

[133] Martinez J.D., Arrieta E., Naranjo A., Monsalve P., Mintz K.J., Peterson J., Arboleda A., Durkee H., Aguilar M.C., Pelaez D. (2021). Rose Bengal Photodynamic Antimicrobial Therapy: A Pilot Safety Study. Cornea.

[134] Amescua G., Arboleda A., Nikpoor N., Durkee H., Relhan N., Aguilar M.C., Flynn H.W., Miller D., Parel J.-M. (2017). Rose Bengal Photodynamic Antimicrobial Therapy: A Novel Treatment for Resistant *Fusarium* Keratitis. Cornea.

[135] Pellegrino F., Carrasco M.A. (2013). Argon Laser Phototherapy in the Treatment of Refractory Fungal Keratitis. Cornea.

[136] Chaurasia S., Mundra J., Dhakal R., Mohamed A., Jha G., Joseph J., Murthy S. (2019). Outcomes of therapeutic penetrating keratoplasty in 198 eyes with fungal keratitis. Indian J. Ophthalmol..

[137] Shi W., Wang T., Xie L., Li S., Gao H., Liu J., Li H. (2010). Risk Factors, Clinical Features, and Outcomes of Recurrent Fungal Keratitis after Corneal Transplantation. Ophthalmology.

[138] Gong Y., Xin M. (2019). Incidence of recurrent fungal keratitis after primary keratoplasty and visual outcome and prognosis after intervention for the recurrence. Medicine.

[139] Lin H.-C., Lin J.-L., Lin-Tan D.-T., Ma H.-K., Chen H.-C. (2012). Early Keratectomy in the Treatment of Moderate *Fusarium* Keratitis. PLoS ONE.

[140] Perry H.D., Doshi S.J., Donnenfeld E.D., Bai G.S. (2002). Topical Cyclosporin A in the Management of Therapeutic Keratoplasty for Mycotic Keratitis. Cornea.

[141] Chatterjee S., Agrawal D. (2021). Use of Topical Cyclosporine 0.1% in Therapeutic Penetrating Keratoplasty for Fungal Keratitis. Cornea.

[142] Onyewu C., Blankenship J., Del Poeta M., Heitman J. (2003). Ergosterol Biosynthesis Inhibitors Become Fungicidal when Combined with Calcineurin Inhibitors against Candida albicans, Candida glabrata, and Candida krusei. Antimicrob. Agents Chemother..

[143] Marchetti O., Moreillon P., Glauser M.P., Bille J., Sanglard D. (2000). Potent Synergism of the Combination of Fluconazole and Cyclosporine in Candida albicans. Antimicrob. Agents Chemother..

