# Supplementary material for: Fusarium Keratitis—Review of Current Treatment Possibilities
Source: J Clin Med. 2021 Nov 23;10(23):5468. doi: 10.3390/jcm10235468 (PMC8658515; doi:10.3390/jcm10235468)

Supplementary materials

Search strategy overview

PubMed

(((("fusarium"[MeSH Terms] OR "fusarium"[All Fields]) AND ("keratitis"[MeSH Terms] OR "keratitis"[All Fields])) OR (("fusarium"[MeSH Terms] OR "fusarium"[All Fields]) AND keratomycosis[All Fields])) OR (mycotic[All Fields] AND ("keratitis"[MeSH Terms] OR "keratitis"[All Fields]))) OR (("microbiology"[MeSH Terms] OR "microbiology"[All Fields] OR "fungal"[All Fields] OR "fungi"[MeSH Terms] OR "fungi"[All Fields]) AND ("keratitis"[MeSH Terms] OR "keratitis"[All Fields]))

Number of results: 9 568

Embase (Elsevier)

'fusarium keratitis'/exp OR 'fusarium keratitis' OR 'keratomycosis'/exp OR keratomycosis OR 'mycotic keratitis'/exp OR 'mycotic keratitis' OR (mycotic AND ('keratitis'/exp OR keratitis)) OR 'fungal keratitis'/exp OR 'fungal keratitis' OR (fungal AND ('keratitis'/exp OR keratitis))

Number of results: 4 154

Cochrane CENTRAL

ID Search

#1 (Fusarium keratitis):ti,ab,kw OR (Fusarium sp): ti,ab,kw

#2 (fungal keratitis):ti,ab,kw OR (corneal inflammation):ti,ab,kw OR ("open angle glauckeratitisoma"): ti,ab,kw

#3 (fusarium keratomycosis): ti,ab,kw OR (Fusarium sp): ti,ab,kw

#4 (mycotic keratitis): ti,ab,kw OR (Fusarium sp): ti,ab,kw

Number of results: 28, 128, 16 and 35 respectively for #1st, #2nd, 3rd and 4th search modalities

In the Cochrane Database we also find 1 systematic review and 123 Clinical Trials protocols

Web of Science

fusarium keratitis (All Fields) or fusarium keratomycosis (All Fields) or fungal keratitis (All Fields) or mycotic keratitis (All Fields)

Refined by: DOCUMENT TYPES: (ARTICLE)

Number of results: 2 956

SCOPUS

(TITLE-ABS-KEY (fungal AND keratitis) OR TITLE-ABS-KEY (fusarium AND keratitis) OR TITLE-ABS-KEY (fusarium AND keratomycosis) OR TITLE-ABS-KEY (mycotic AND keratitis))

Number of results: 3 538

Supplementary materials

Additionally we searched the Clinical Trials registers websites available via institutional access of Wrocław Medical University

Additionally we concluded the possible source to be from previous systematic reviews and meta-analysis studies we found 328 studies for this criterion.

PICO statement :

1) Patient: Included only patients that have been diagnosed with *Fusarium* keratitis with microbiologic confirmation.
2) Intervention: All types of medication were included from topical, through intracameral to systemic therapy.
3) Comparison: We intend to compare each possible treatment strategy to reveal the most applicable and find if there is any "special situations" that require further concern and change of standard procedure approach.
4) Outcome: Treatment that has been successful in the early and late stage of *Fusarium* keratitis in either reduction of rate of enucleation or corneal transplant; or in improvement of best-corrected visual acuity after the recovery. An additional factor primarily intended were reduction of hospitalisation time and/or close careful ophthalmologic care (some Departments prefer the outpatient "each day close follow-up" strategy) but due to the scarce resources this was only mentioned in the review.

Supplementary materials

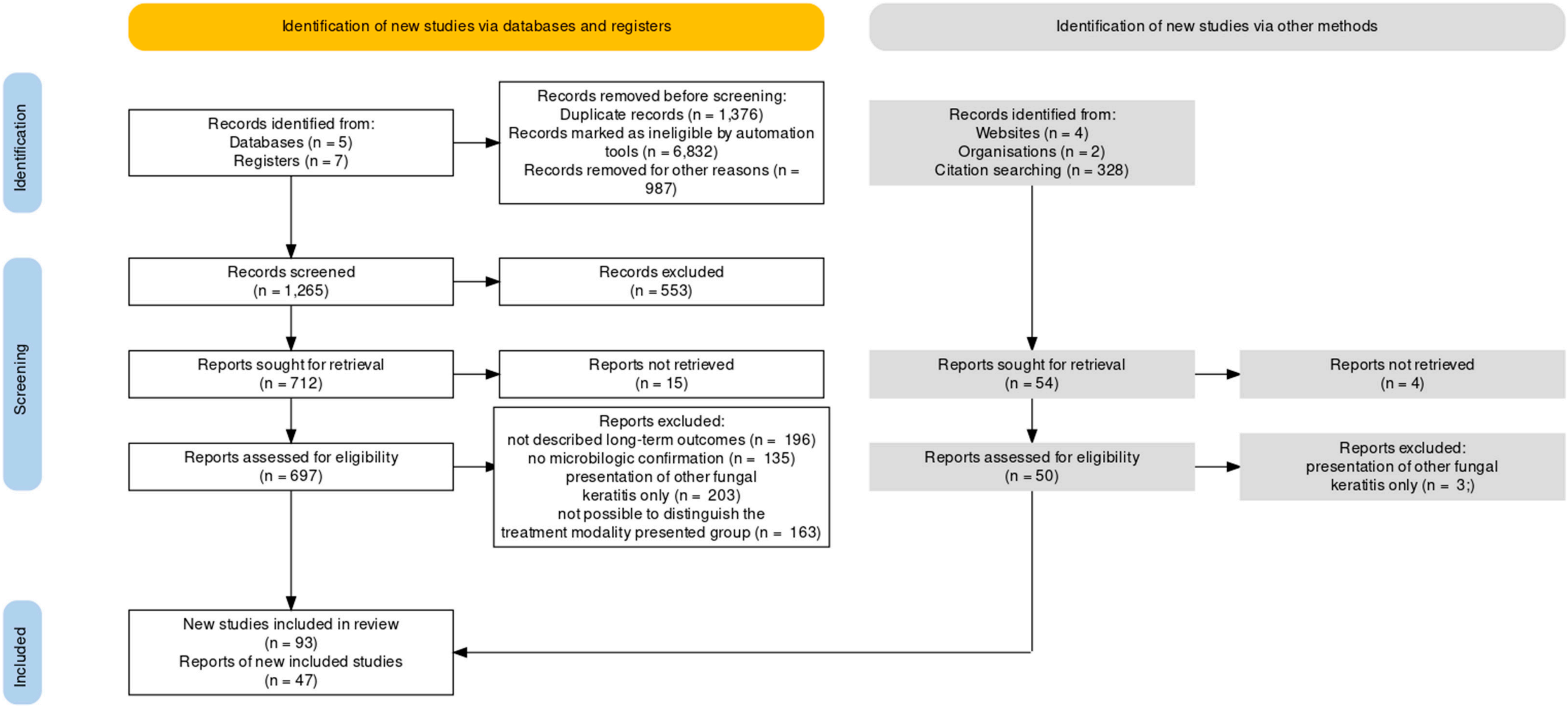

Supplement: Supplementary file 1 [file jcm-10-05468-s001.zip › jcm-1447181-supplementary.pdf]
